# Erythropoietin and Protection of Renal function in Cardiac Surgery (EPRICS) trial

**DOI:** 10.1186/cc13574

**Published:** 2014-03-17

**Authors:** A Dardashti

**Affiliations:** 1 Lund University and Skane University Hospital, Lund, Sweden

## Introduction

To date, there are no known methods for preventing acute kidney injury after cardiac surgery. Increasing evidence suggests that erythropoietin (EPO) has renal anti-apoptotic and tissue protective effects [1], and in animal models a single high dose of EPO has been shown to ameliorate reperfusion injury after ischemia [2]. However, recent human studies have shown conflicting results. We aimed to study the effect of a single high dose of EPO preoperatively on renal function after coronary artery bypass grafting (CABG) in patients with preoperative impaired renal function.

## Methods

This single-centre, randomized, double-blind, placebocontrolled study included 75 patients scheduled for CABG with preexisting renal impairment (estimated glomerular filtration rate based on p-cystatin C <60 ml/minute and >15 ml/minute). The patients either received a single high dose of EPO (400 lU/kg) or placebo preoperatively. The primary endpoint was renal protection evaluated by p-cystatin C at the third postoperative day compared with the preoperative values. Incidence of acute kidney injury and other renal biomarker changes were among secondary endpoints.

## Results

There was no significant difference on the third postoperative day for p-cystatin C levels (2.1 ± 0.8 mg/l for the study group and 1.9 ± 0.5 mg/l for the control group, *P *= 0.51). There were no significant differences in other renal biomarkers or measures between the groups (p-NGAL, p-creatinine, p-urea, and estimated glomerular filtration rate). There were no other differences in outcome variables between the groups.

## Conclusion

Intravenous administration of a single high dose (400 IU/ kg) of EPO did not have a renal protective effect in patients with reduced kidney function undergoing coronary artery bypass surgery.

## References

[B1] Bahlmann FH Fliser D Erythropoietin and renoprotection Curr Opin Nephrol Hypertens 2009 18 15 20 10.1097/MNH.0b013e32831a9dde 19077684

[B2] Moore E Bellomo R Erythropoietin (EPO) in acute kidney injury Ann Intensive Care 2011 1 3 10.1186/2110-5820-1-3 21906325 PMC3159901

